# XBB: the chosen one from SARS-CoV-2 homologous recombination

**DOI:** 10.1093/lifemedi/lnae002

**Published:** 2024-02-13

**Authors:** Qi Chen, Pan-Deng Shi, Cheng-Feng Qin

**Affiliations:** Department of Virology, State Key Laboratory of Pathogen and Biosecurity, Beijing Institute of Microbiology and Epidemiology, AMMS, Beijing 100071, China; Department of Virology, State Key Laboratory of Pathogen and Biosecurity, Beijing Institute of Microbiology and Epidemiology, AMMS, Beijing 100071, China; Department of Virology, State Key Laboratory of Pathogen and Biosecurity, Beijing Institute of Microbiology and Epidemiology, AMMS, Beijing 100071, China

In the early stage of the COVID-19 pandemic, the emergence of SARS-CoV-2 variants was mainly led by the accumulation of amino acid mutations, such as the emergence of the known variants of concern, including Alpha, Beta, Delta, to Omicron. With the emergence of more variants, co-circulation of different SARS-CoV-2 viral strains appeared in many regions, which inevitably led to co-infection. Of note, SARS-CoV-2 recombination signs were quickly found in some co-infections, including the recombination occurred between different variants, such as Delta and Omicron variants, and the recombination occurred between subvariants, such as Omicron BA.1 and BA.2. Genetic evolution analysis indicated that around 2.7% of sequenced SARS-CoV-2 genomes have detectable recombinant ancestry [1]. Luckily, most of these recombinants did not become predominant circulating strains and disappeared rapidly. However, things changed since the summer of 2022: the newly appeared recombinant XBB (also known as BA.2.10) quickly swept the world and caused new waves of epidemics worldwide.

**Figure 1.**
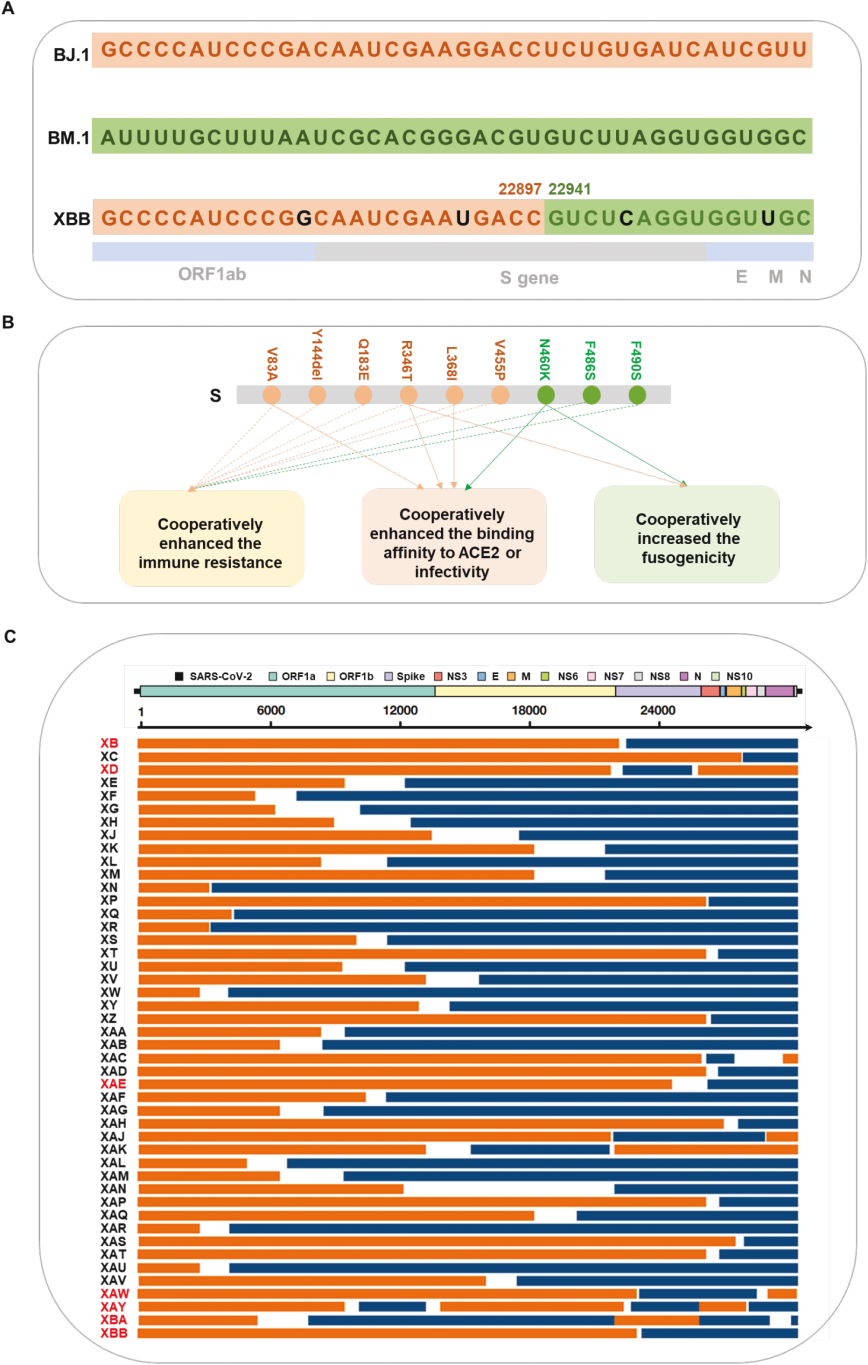
**Recombination characteristics of SARS-CoV-2 recombinants and the effects of the rearranged substitutions for the changes of viral characteristics.** (A) Nucleotide differences between the consensus sequences of the BJ.1, BM.1 (including BM.1.1/BM.1.1.1) lineages and the XBB (including XBB.1) lineage and the recombination breakpoint. (B) The rearranged substitutions in the S led by recombination cooperatively enhanced immune resistance, binding affinity, infectivity, and fusogenicity of XBB. (C) Schematic of the breakpoints of SARS‐CoV‐2 recombinants. Names of recombinants with breakpoints located in the S gene are specially indicated in red. The recombinants were visualized according to the results published in pango-designation, and the parent lineages and breakpoints were drawn with reference to the data in github.com/cov-lineages/pango-designation/issues.

The XBB variant was first noted by Cornelius Roemer from the University of Basel on 13 September 2022, from the sequences uploaded to GISAID by Singapore on the same day (github.com/cov-lineages/pango-designation/issue). Based on unique mutations found in the genome, XBB was proposed to be a potential recombinant between two BA.2 descendants, BJ.1 (BA.2.10.1.1) and BM.1.1.1 (BA.2.75.3.1.1.1; a descendant of BA.2.75). Subsequently, a team led by Professor Kei Sato from the University of Tokyo’s Institute of Medical Science systematically analyzed the characteristics of the XBB variant [2]. To trace the evolution of the XBB variant, the maximum likelihood tree of representative sequences from PANGO lineages of interest: BA.1, BA.2, BA.4, BA.5, BA.2.75, BJ.1, and BM.1.1.1, rooted on a B.1.1 outgroup was built, which clearly showed that the XBB variant emerged as a unique lineage between the second-generation BA.2 variants BJ.1 and BM.1.1.1. In addition, visual inspection of the nucleotide differences between the consensus sequences of XBB, BJ.1, and BM.1 illustrated that the identity of XBB to BJ.1 ends at genome position 22,942, and the identity of XBB to BM.1 starts after position 22,896 (Fig. 1A). Further analysis with different independent recombination detection methods implemented in Recombination Detection Program (v.5.21) (RDP5) identified a single recombination breakpoint unique to all XBB sequences at genomic position 22,920, which is consistent with the result of visual inspection. These findings collectively indicated that the XBB variant is a recombinant occurred between BJ.1 and BM.1.1.1 with the recombination breakpoint between positions 22,897 and 22,941. Notably, the breakpoint is just located in the receptor binding domain (RBD) (corresponding to amino acid positions 445–460), and the recombination led to the rearrangement of substitutions in the Spike (S) (Fig. 1B).

XBB and its descendants have aroused global concern due to its superior transmissibility and significant immune escape. Masaki Imai et al. recently found that the therapeutic monoclonal antibodies, including REGN10987 (marketed as imdevimab), REGN10933 (marketed as casirivimab), COV2-2196 (marketed as tixagevimab), COV2-2130 (marketed as cilgavimab), and S309 (the precursor of sotrovimab), did not neutralize XBB isolates even at the highest 50% focus reduction neutralization test (FRNT50) value (> 50,000 ng/mL) [3]. In addition, the XBB was also confirmed much more immunoevasive than the ancestral strain and BA.5.2 to the sera from individuals who received CoronaVac (Sinovac) and BNT16b2 (Pfizer-BioNtech) [4]. In fact, even the sera from vaccinated individuals followed by BA.5 infection are still at a low level of neutralization against XBB lineages [5]. Further analysis revealed that substitutions in the XBB S obtained from its parental variants cooperatively contribute to the immune resistance [2]. For instance, BM.1-derived F486S and F490S and BJ.1-derived V83A, Y144del, Q183E, R346T, L368I, and V445P cooperatively contribute to the resistance against humoral immunity induced by breakthrough BA.2 infection, and the BJ.1-derived V445P and BM.1-derived N460K cooperatively contributed to escape from humoral immunity elicited by vaccination (Fig. 1B). These observations suggest that recombination has made XBB antigenically different from the other Omicron subvariants because of the rearrangement of substitutions in the S, and, therefore, markedly evades previous infection and vaccination-induced herd immunity in the human population.

Apart from the immune resistance, the binding affinity, infectivity, and fusogenicity of the XBB were also increased by recombination [2]. With a yeast surface display assay, it was found that the binding affinity of XBB.1 S RBD to human ACE2 receptor is significantly higher than that of ancestral BA.2 S RBD, which is attributed to the simultaneously obtained substitutions BM.1-derived N460K and BJ.1-derived R346T and L368I by recombination [2]. Consistent with the increased binding affinity, the XBB.1 S augments its infectious potential for HOS-ACE2/TMPRSS2 cells through the cooperation of multiple substitutions in the S, including the BJ.1-derived V83A, R346T, and L368I and the BM.1.1.1-derived N460K (Fig. 1B) [2]. Furthermore, the simultaneously obtained substitutions (R346T and N460K) in the RBD of XBB.1 S by recombination also increased the fusogenicity of XBB.1 (Fig. 1B) [2]. These findings confirmed that recombination also has the potential to change the infectivity and even pathogenicity in addition to the immune escape.

The viral characteristic changes of XBB caused by recombination finally led to increased viral fitness [2]. First of all, the *R*_e_ value of XBB was found higher than that of its parental BJ.1 and BM.1.1.1, respectively. In addition, the *R*_e_ value of XBB was also found higher than the cocirculated variant, such as BA.5, in Singapore and other countries. Notably, this is the first documented example of a SARS-CoV-2 variant increasing its fitness through recombination rather than substitutions, which may also give an explanation as to why the XBB finally outcompeted other variants and dominated this country. In fact, if we look deep into all SARS-CoV-2 genomes, we will find that more than 45 SARS-CoV-2 recombinants have been defined before XBB (Fig. 1C) (github.com/cov-lineages). Despite the XBB variant (including its sublineages) is the only one among so many recombinants that finally dominated the whole world, which can be regarded as the chosen one from homologous recombination, it clearly demonstrated the success of the evolution strategy of recombination for SARS-CoV-2. Meanwhile, we also need to look out for two facts: one is that the recombination breakpoints preferentially occur in the 3ʹ portion of the genome, especially in the S gene [1], and the recombination breakpoints of 7 of the 46 SARS-CoV-2 recombinants appeared till XBB are located in the S (Fig. 1C); the other is that new SARS-CoV-2 recombinants are still emerging after XBB appearance (github.com/cov-lineages). These facts remind us that new recombinants with breakpoints and rearranged substitutions in S are probably to occur in the future. Thus, we can learn that the XBB may be the chosen one from homologous recombination for the current stage, but may not be the only one in the future, which clearly indicates that we should keep alert to the evolution strategy of recombination of SARS-CoV-2, and the continued viral genomic surveillance and real-time evaluation of the risk of newly emerging SARS-CoV-2 recombinants is especially necessary.
